# Biofilms and lichens on stone monuments: do they damage or protect?

**DOI:** 10.3389/fmicb.2014.00133

**Published:** 2014-04-02

**Authors:** Daniela Pinna

**Affiliations:** Soprintendenza per i Beni Storici e Artistici, Scientific LaboratoryBologna, Italy

**Keywords:** biofilms, lichens, stone monuments, biogenic weathering, protection, treatments

With this paper, I focus on a topic that, in my opinion, is worth consideration by scientific community involved in the conservation of cultural heritage. As far the role that biofilms and lichens play in the weathering of natural and artificial stones, an increasing number of researches account for a negligible effect and even for a protection. Thus, the axiomatic correlation among biofilms, lichens, and stone weathering is matter of controversy. These researches bring a novel perspective in a field where many studies showed that biofilms and lichens do damage stones. In this paper, I report some results that in my opinion add significant contributions and useful information to the subject.

The microflora of outdoor stone monuments represents a complex ecosystem including bacteria, algae, fungi, lichens. Microorganisms colonize stonework whenever the conditions of moisture, light, temperature, and nutrition are favorable. When a biological colonization is evident, the conservator should verify at which extent it damages the materials and know the non-biogenic agents that take part in the degradation (Warscheid, 2003). Many causes have similar effects, act in synergy, or interact in quantitatively variable relations. Thus, the relevance of biological impact to the entire deterioration process should be evaluated very carefully. Detecting microorganisms on heritage objects does not automatically imply that they actually change the chemical composition or physical properties of the materials. A study of different kind of sandstone from Wyoming covered with lichens and old petroglyphs (Chiari and Cossio, 2002) demonstrated for example that lichens were not one of the key factors in the conservation of the petroglyphs, either in a negative (destruction of the outermost layer) or in a positive way (protection from rain, sun, etc.). Lichens filled the gaps between the grains, which were large enough to host them without exercising relevant pressure. Porosity was less toward the outside, since in the outer layer lichens occluded the pores. Counting the lichen thallus, the porosity proved to be the same as in the core of the rock. The deterioration of the sandstone depended mainly on the nature of the sandstone itself, in particular on the dimension of the quartz grains: the larger the grains, the greater the porosity, water absorption, fragility, and de-cohesion of the sandstone. The results may help decide whether to eliminate the lichens from the surface and to account the need for their removal to aesthetic or site management reasons rather than to chemical-physical reasons.

Another stimulating study investigated the interaction over time among biofilms, lichens, and sandstone, and compared the behavior of siliceous rocks with that of carbonate ones in relation to biological growth (Hoppert et al., 2004). The authors hypothesized that lithobiontic organisms temporarily stabilize loosely to moderately cemented sandstones. When complex biofilms and lichens with moderate metabolic and reproductive rates colonize the rock in high densities, prevention of rapid decomposition of the stone is necessary. To enable undisturbed growth over several years or even decades, complex microbial communities and lichens protect the substratum from rapid decomposition by formation of a tight network of cells and extracellular polymers, which surround the mineral particles (e.g., quartz). Enwrapping the grains with a biogenic matrix temporarily stabilizes the surface and reduces weathering, which may allow the organisms to persist for years. On the contrary, microorganisms contrive a different pattern of growth in homogeneous carbonate rocks, actively boring cavities without regard of the pre-existing rock-fabric. However, a structural weakening of the substratum involving the risk of sudden desquamation and destruction of the endolithic environment create by microorganisms is uncommon. Thus, on carbonate substrata, endoliths with their relatively slow growth rates (compared to epiliths) have a chance for a sustainable life for long periods. The authors deduced that, on homogeneous carbonate substrata, maintenance of a stable population for tens of years accounts for a “sustainable” use of the rock substratum. They suggested it is a special feature of lichens that develop over decades. Understanding their strategies for colonization may be important for a variety of aspects concerning biogenic stone deterioration.

A survey of four sandstone heritage structures in central Belfast exposed for around 100 years and colonized by green algae biofilms (Cutler et al., 2013) showed that algal patches were associated with less weathered surfaces (i.e., harder algal patches were associated with lower coefficient of variation of surface hardness). This might indicate that green algal cover had a broadly bio-protective role.

Results from the comparative analysis of the water transport data on a temple located in Angkor Wat (Cambodia) suggested that lichens had an important moisture-controlling function for the environmentally stressed stones (Warscheid and Leisen, 2011). The measurement of the capillary water uptake at different places on the temple showed that lichens protected the stone from rapid water uptake, whereas certain algal and blackened cyanobacteria biofilms significantly increased it. Rebound hardness and drill resistance measurements taken on lichens, did not show any evidence that they significantly affected the mechanical properties of the stones. Consequently, the authors stated that the lichens regulated the humidity, thermal transmission, and water vapor diffusion, reducing thermo-hygric stresses to the stone.

Under conditions of high abiotic weathering, lichens can provide protection from wind and rain to the stone surface (Carballal et al., 2001; Bungartz et al., 2004), or limit erosion by reducing the level of water within the rock (Garcia-Vallès et al., 2003). Their retention of moisture within the thallus reduced thermal stress on a limestone surface (Carter and Viles, 2003). According to Ariño et al. (1995) and Wendler and Prasartet (1999), lichens can have a protective effect against the natural decay of porous stones, by decreasing either the intensity of water exchanges between the substrate and the environment, or the damage by atmospheric agents (wind, rain, pollutants, salt aerosol). More widespread exfoliation, saline efflorescences, flaking, and honeycombing of non-colonized surface supported this hypothesis.

All the quoted papers give relevant suggestions to deepen the knowledge of the role of biofilms and lichens in conservation of stone. The measurements of the capillary water uptake and the surface hardness of colonized and non-colonized materials, the drill resistance measurements taken on lichens, the measurements of the surface porosity of a colonized stone compared with that of the core of the rock, are scientific methods suitable to develop the studies on the interaction between microflora and stones. Moreover, the knowledge of biofilms strategies for colonization would be relevant to understand whether they are capable to stabilize poorly cemented stones. As the literature concerning the degradation of stone by lichens is copious, well documented and has experimentally proved, any confutation must provide an equal weight of proof in order to be considered valid (Piervittori et al., 2004). Many authors showed that lichens are indeed generally defacing and intrinsically damaging. Moreover, the decay of the lichen thallus, which occurs on the center of the colonies of some species, can open the underlying area to further weathering, resulting in cratered mounts on the rock surface (Mottershead and Lucas, 2000). Although the protective effects of lichens deserve further research, this aspect cannot be generalized, and each case should be examined on its own merits.

Despite the numerous literature dealing with the damage of the materials colonized by biofilms (see reviews in Caneva et al., 2008 and Scheerer et al., 2009), the relationship between mineral solubility and the role of microbial surface colonization in weathering reactions is a topic that has yet to be answered in a comprehensive manner (Davis and Luttge, 2005). The precise role of mineral solubility, and hence dissolution rate, in determining the extent and rate of microbial surface colonization is largely unknown. Moreover, the relative importance of various weathering mechanisms as a function of species composition, rock type, and external conditions (moisture, temperature, pollution, and climate) needs further studies.

An interesting work (Gutarowska and Zakowska, 2002) deals with a very important topic of research that is the quantification of risk by fungal growth on surfaces. Although the work did not focus on materials of cultural heritage, it can be relevant all the same. It correlated the amount of ergosterol to the CFU number of fungi inoculated on samples made of concrete, gypsum board, emulsion coat, brick, and plaster. Ergosterol is a basic sterol of cellular membranes in filamentous fungi and yeast. Ergosterol content lower than 2.12 mg/m^2^ corresponds to the normal level of spores contamination without any active growth; values ranging 2.12–3.96 mg/m^2^ indicate the activation of mycelium growth, and those over 3.96 mg/m^2^ reveal active fungal growth and high contamination. Based on them it could be possible to estimate the level of fungal contamination of materials, also when the mycelium is inactive and cannot be detected using traditional methods.

The role of lichens in stone weathering strictly related to the removal treatments. A few recent articles are a plea for greater consideration when treating exterior stone covered in lichens. Although their removal from tombstones, sculptures, and monuments is widely practiced, it can damage the stone, and, in the case of extensive and repeated use of biocides, the environment. For example, the mechanical removal of crustose lichens is particularly difficult because the thallus forms an intimate association with the substrate. Hence, its removal leads to severe structural damage (Scheerer et al., 2009). Rather than resort to mechanical, or biocidal cleaning, all of which have major disadvantages, Sheppard (2007) favors minimal intervention proposing non-destructive documenting/recording of the monuments and letting the lichens contribute to the aesthetics of churchyards and cemeteries. Moreover, certain forms of biological growth can have a scarcity or rarity that must be taken into account when planning conservation work (Watt, 2006). Where churchyards are concerned, there may also be a preference for conserving bio-diversity, including ferns and lichens on monuments.

In conclusion, I think that the scientific community involved in this field is mature enough to go beyond the studies reporting just unending lists of species. In my opinion, the assessment of a “common language,” of standard test methods, for the evaluation of the damage by biofilms and lichens (see as an example the paper by Gutarowska and Zakowska) is very important. Standard test procedures are essential to compare the results of different laboratories, and to interpret, understand, and evaluate the research. The application of standard procedures will ultimately result in proposing indexes of risk or danger of biofilms and lichens on different stones.

## Conflict of interest statement

The authors declare that the research was conducted in the absence of any commercial or financial relationships that could be construed as a potential conflict of interest.
